# Intraoperative vasopressor use and early postoperative acute kidney injury in elderly patients undergoing elective noncardiac surgery

**DOI:** 10.1080/0886022X.2022.2061997

**Published:** 2022-04-10

**Authors:** Dilshan Ariyarathna, Ajinkya Bhonsle, Joseph Nim, Colin K. L. Huang, Gabriella H. Wong, Nicholle Sim, Joy Hong, Kirrolos Nan, Andy K. H. Lim

**Affiliations:** a Department of General Medicine, Monash Health, Clayton, Victoria, Australia; b Department of Nephrology, Monash Health, Clayton, Victoria, Australia; c Department of Medicine, School of Clinical Sciences, Monash University, Clayton, Victoria, Australia

**Keywords:** Geriatrics, surgical procedures, operative, hypotension, vasoconstrictor agents, acute kidney injury

## Abstract

**Background:**

Intraoperative hypotension is a risk factor for postoperative acute kidney injury (AKI). Elderly patients are susceptible due to reduced responses to acute hemodynamic changes.

**Aims:**

Determine the association between hypotension identified from anesthetic charts and postoperative AKI in elderly patients.

**Methods:**

Retrospective cohort study of elective noncardiac surgery patients ≥65 years, at an Australian tertiary hospital (December 2019–March 2021), with the primary outcome of AKI ≤48 h of surgery. Factors of interest were intraoperative hypotension determined from anesthetic charts (mean arterial pressure <60 mmHg, systolic blood pressure <90 mmHg, recorded 5-min) and intraoperative vasopressor use.

**Results:**

In 830 patients (mean age 75 years), systolic hypotension was more frequent than mean arterial hypotension (25.7% vs. 11.9%). Most hypotensive episodes were brief (7.2% of systolic and 4.2% of mean arterial hypotension lasted >10 min) but vasopressors were used in 84.7% of cases. The incidence of postoperative AKI was 13.9%. Systolic hypotension >20 min was associated with AKI (OR, 3.88; 95% CI: 1.38–10.9), which was not significant after adjusting for vasopressors, creatinine, American Society of Anesthesiologists class, and hemoglobin drop. The cumulative dose of any specific vasopressor >20 mg (or >10 mg epinephrine) was independently associated with AKI (adjusted OR, 2.47; 95% CI: 1.34–4.58). Every 5 mg increase in the total dose of all intraoperative vasopressors used during surgery was associated with 11% increased odds of AKI (95% CI: 3–19%).

**Conclusions:**

High vasopressor use was associated with postoperative AKI in elderly patients undergoing noncardiac surgery, independent of hypotension identified from anesthetic charts.

## Introduction

Intraoperative hypotension is a common complication of general anesthesia and surgery which is associated with postoperative acute kidney injury (AKI) [1,2]. The development of AKI has implications for morbidity and mortality. In the short term, postoperative AKI is associated with greater postoperative complications, longer hospital and intensive care unit length of stay, higher healthcare costs, and greater readmission rates at 30 days [3,4]. In the longer term, postoperative AKI is associated with an adjusted hazard ratio of death of 1.20 (95% CI, 1.10–1.30) [5]. AKI is a well-established risk for chronic kidney disease (CKD), and even mild AKI is associated with long-term kidney impairment [6].

Elderly patients may be more at risk of intraoperative hypotension and AKI due to a number of age-related changes in cardiovascular physiology. These include endothelial dysfunction, increased arterial stiffness, reduced left ventricular compliance and cardiac reserve, and impaired β-adrenergic and parasympathetic function [7]. The blunted response to vasodilation from anesthesia and hypovolemia from blood loss, could contribute to postoperative AKI. Conversely, patients undergoing elective surgery may have a lower risk of hypotension and AKI compared to patients undergoing emergency surgery, and this group of patients have been less intensively studied.

The aims of this study were to determine the incidence of early postoperative AKI in elderly patients after elective noncardiac surgery and the association between AKI and intraoperative hypotension or vasopressor/inotrope support. We hypothesized that evidence of intraoperative hypotension and vasopressor or inotrope use obtained from standard anesthetic charts could be used to predict postoperative AKI in the initial 48 h after surgery.

## Methods

### Study design, setting, and ethics approval

We conducted a retrospective cohort study of elective noncardiac surgery patients at three major metropolitan hospitals in the Monash Health Network in the state of Victoria, Australia (Monash Medical Centre, Dandenong Hospital, and Casey Hospital), from December 2019 to March 2021. This study was approved by the Monash Health Human Research Ethics Committee as a Quality Initiative as all data were obtained from standard medical documentation and laboratory results generated during routine clinical practice, and the requirement for patient consent was waived (Monash HREC reference RES-21-0000077Q-73251, ERM reference 73251).

### Participants

Participants were identified from the network database of elective admissions during the study period. Our study inclusion criteria were (1) age ≥65 years, (2) hospital length of stay ≥48 h, which is the time required to diagnose postoperative AKI, and (3) noncardiac surgery, but also excluding obstetric and ophthalmology. Our study exclusions were patients who (1) did not require a general anesthetic, (2) had missing or inadequate hemodynamic data, (3) had missing baseline or postoperative serum creatinine measurement, (4) are receiving chronic dialysis therapies.

### Acute kidney injury

The primary outcome of this study was the development of AKI of any stage (severity) within 48 h postoperatively. We used the Kidney Disease: Improving Global Outcomes (KDIGO) creatinine criteria to define and describe the severity of AKI: Stage 1 is a serum creatinine increase of ≥0.3 mg/dL (27 µmol/L) within 48 h, or increase ≥1.5 times baseline; Stage 2 is a serum creatinine increase 2.0–2.9 times baseline; and Stage 3 is a serum creatinine increase 3.0 times baseline, increase to ≥4.0 mg/dL (≥354 µmol/L), or initiation of renal replacement therapy [8]. Urine output criteria for AKI were not used as it was not uniformly recorded for all patients. In order of preference depending on availability, the baseline serum creatinine was estimated by (1) taking the admission or preadmission serum creatinine value, (2) calculating the mean values from stable outpatient tests within the last 12 months, or (3) taking the final creatinine measurement before discharge from a prior admission within 12 months. In this study, we restricted the follow-up to 48 h postoperatively, to ensure the minimum time for a diagnosis of AKI was achieved, while minimizing confounding by other postoperative complications such as sepsis.

### Intraoperative hypotension

Intraoperative hypotension was defined as a mean arterial pressure (MAP) <60 mmHg or a systolic blood pressure (SBP) <90 mmHg. Baseline blood pressures were obtained from the preoperative and pre-induction readings. Intraoperative blood pressures were obtained from the anesthetic chart, which were documented at 5-min intervals. Most patients received standard noninvasive assessment of blood pressure, and only 2% of patients received preemptive insertion of an intra-arterial catheter for continuous measurements prior to surgery commencement. Due to the limited number of patients receiving invasive monitoring, we did not analyze these patients separately. We determined the number and duration of hypotensive episodes and calculated the cumulative duration of intraoperative hypotension. Postoperatively, blood pressure readings were examined for evidence of hypotension for 24 h. In conjunction with blood pressure readings, we determined the cumulative dose of vasopressor agents used intraoperatively and noted if hemodynamic support were used postoperatively in the intensive care unit. A high dose of any specific vasopressor used was defined as a cumulative dose of metaraminol, ephedrine, phenylephrine, or norepinephrine >20 mg, or a cumulative dose of epinephrine >10 mg, over the duration of surgery (not time-averaged).

### Other variables

We collected data on age, sex, comorbidities, body mass index, surgery type, surgery duration, and the American Society of Anesthesiologists physical status (ASA) classification. The ASA offers a simple classification for assessing perioperative clinical risk, which can be easily communicated for risk-benefit assessment. The assignment of ASA class is made by the anesthetist on the day of surgery based on symptoms, comorbidities, and functional limitations, and it is accepted standard practice in preoperative assessments. Although there are six status classes in this system, only ASA classes 1 to 4 were relevant to this study: ASA 1 (normal healthy patient), ASA 2 (mild systemic disease), ASA 3 (severe systemic disease), ASA 4 (severe systemic disease that is a constant threat to life), ASA 5 (moribund patient not expected to survive without the operation), ASA 6 (brain-dead organ donor). A full description with examples can be found on the ASA website [9].

From the medical records and medication charts, we also determined the baseline use of β-blockers, renin-angiotensin system inhibitors and diuretics, exposure to potential nephrotoxic medications (aminoglycosides, vancomycin, nonsteroidal anti-inflammatory drugs) within 24 h pre-operatively and postoperatively, and exposure to intravenous or intra-arterial iodinated contrast within 72 h preoperatively. We determined the postoperative hemoglobin as the nadir within 48 h of surgery and calculated the drop in hemoglobin compared to preoperative levels. The hemoglobin drop was corroborated with any intraoperative red blood cell transfusions, and transfusions within 24 h after surgery. Based on the established baseline serum creatinine, chronic kidney disease (CKD) was defined as an estimated glomerular filtration rate (eGFR) <60 mL/min/1.73 m^2^ using the CKD Epidemiology Collaboration (CKD-EPI) equation [10].

### Statistical analysis

Normally distributed continuous variables were described using mean and standard deviation (*SD*), while the distribution of skewed data was described with the median and interquartile range (IQR). Categorical data analysis was performed using the chi-squared test (*χ*^2^) or Fisher’s exact test. Initial univariable comparisons of continuous data was conducted with a *t*-test or nonparametric Wilcoxon rank sum test. For the regression of vasopressor use on the first SBP reading, we used a zero-inflated negative binomial approach as not all patients developed hypotension and needed vasopressors. We used logistic regression (*purposeful selection* method) to determine the association between AKI and the variables of interests (blood pressure, vasopressor use) while adjusting for covariates. All clinically relevant and statistically significant covariates (*p* < .20) were entered into a base model. This was followed by backwards elimination and a stepwise approach, to retain variables with a *p* < .05 or significantly confounded the association between hypotension and AKI (change in *b*-coefficient >10%). We used Akaike’s and Bayesian information criteria or likelihood ratio tests to compare model fit. We assessed for collinearity by examining the strength of the correlation between variables and estimated the variance inflation factor. Model diagnostics included analysis of model residuals, goodness-of-fit, outliers, and influential observations. Analyses were performed using STATA 16.1 (StataCorp, TX, USA). A *p* < .05 was considered statistically significant.

## Results

### Patient characteristics

**Figure 1.**
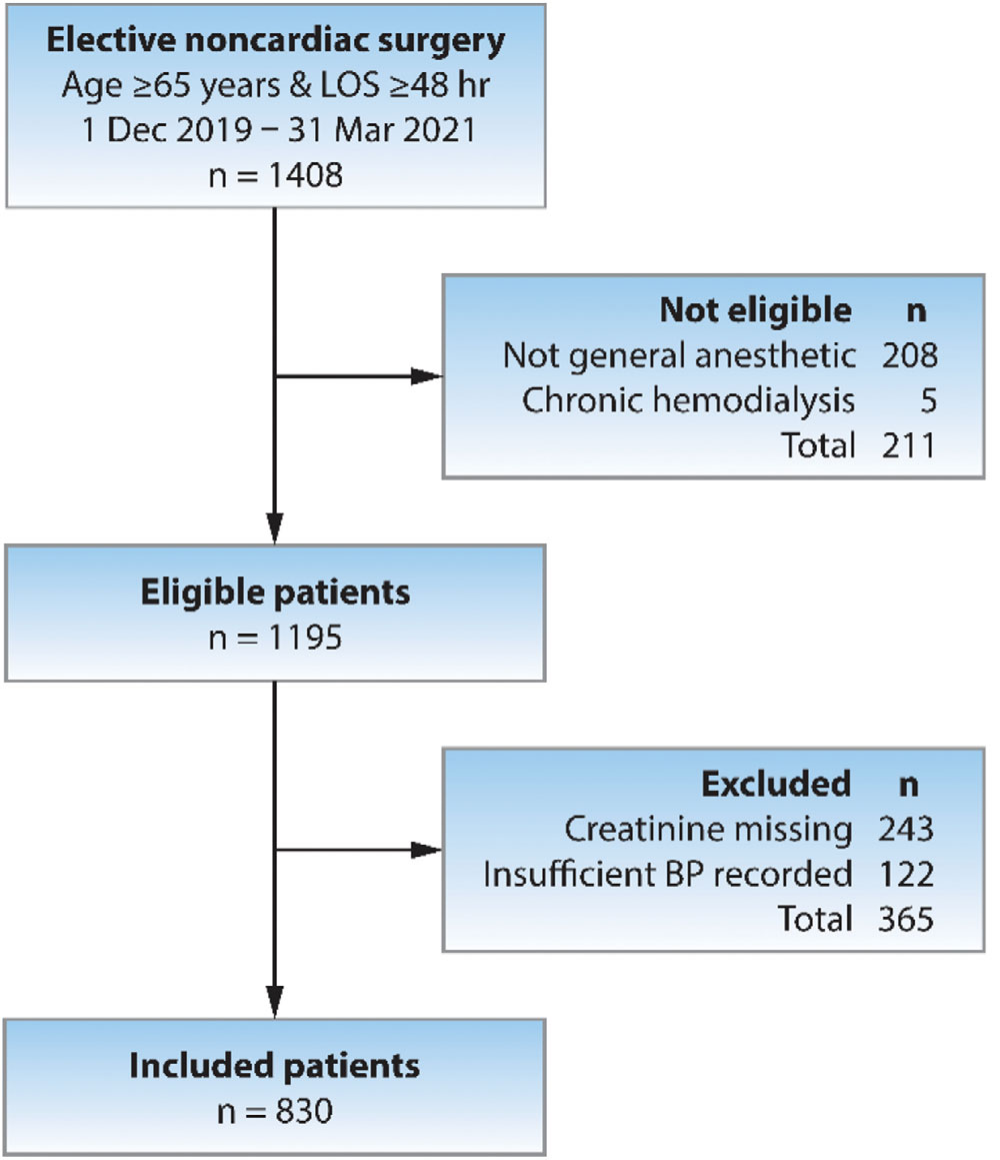
Study flow diagram showing patient eligibility and exclusions. LOS: hospital length of stay; BP: blood pressure.

We included a total of 830 patients aged ≥65 years in the final analysis as shown in the study flowchart (Figure 1). The mean age of patients was 74.5 years with approximately equal sex distribution. Overall, 34% of patients were obese and 68% had an ASA class >2. In our patient cohort, chronic hypertension was prevalent (72%), and diabetes affected 28% of patients. The major cardiovascular and respiratory comorbidities of the study population are shown in Table 1. Our study prevalence of heart failure of 6.9% was consistent with that reported for people 60- to 86-year old in Australia from the Canberra Heart Study of 6.7% (95% CI: 4.4–7.1%) [11]. The percentage of patients associated with each method used to determine baseline serum creatinine were: admission or preadmission clinic <1 week prior to admission (40%), average of outpatient creatinine values over recent 12 months (59%), creatinine on previous hospital discharge within the last 12 months (1%). Accordingly, 24.9% of patients had evidence of CKD (eGFR < 60 mL/min/1.73 m^2^).

**Table 1.** Baseline patient characteristics by acute kidney injury status.

Characteristic	All patients (*N* = 830)	No AKI (*n* = 715)	AKI (*n* = 115)	*p* Value
Age, mean (*SD*), years	74.5 (6.6)	74.5 (6.7)	74.9 (6.3)	.55
Male, *n* (%)	447 (53.9)	379 (53.1)	68 (59.1)	.22
Body mass index, mean (*SD*), kg/m^2^	28.4 (5.7)	28.4 (5.7)	28.8 (5.8)	.40
Obese, BMI ≥ 30 kg/m^2^, *n* (%)	278 (34.0)	243 (34.5)	35 (30.7)	.43
ASA physical status classification, *n* (%)				
Class 1	10 (1.2)	8 (1.1)	2 (1.7)	.001
Class 2	258 (31.1)	237 (33.2)	21 (18.3)	
Class 3	505 (60.8)	428 (59.9)	77 (67.0)	
Class 4	57 (6.9)	42 (5.9)	15 (13.0)	
Diabetes, *n* (%)	231 (27.8)	194 (27.1)	37 (32.2)	.26
Heart failure, *n* (%)	57 (6.9)	42 (5.9)	15 (13.0)	.005
Coronary heart disease, *n* (%)	177 (21.3)	140 (19.6)	37 (32.2)	.002
Chronic obstructive pulmonary disease, *n* (%)	115 (13.9)	90 (12.6)	25 (21.7)	.008
Chronic kidney disease, *n* (%)	207 (24.9)	158 (22.1)	49 (42.6)	<.001
Peripheral vascular disease, *n* (%)	97 (11.7)	76 (10.6)	21 (18.3)	.018
Chronic hypertension, *n* (%)	599 (72.2)	503 (70.4)	96 (83.5)	.004

ASA: American Society of Anesthesiologists; BMI: body mass index, missing = 12 (1.4%).

### Incidence of acute kidney injury

The incidence of postoperative AKI was 13.9% (115 of 830 patients). Of patients experiencing AKI, 84.4% were stage 1 (97 of 115 patients), 4.4% were stage 2 (5 of 115 patients), and 11.3% were stage 3 (13 of 115 patients). There were significant differences in the incidence of AKI between surgical units, with the highest incidence noted in general surgery (30.2%), urology (26.7%), vascular (17.2%), and orthopedic (16.4%). The surgical units with a low incidence of AKI (<5%) were neurosurgery (including neuro-interventional radiology), plastics (including breast), faciomaxillary, otolaryngology, and thyroid, noncardiac thoracic surgery, and gynecology. There were some baseline differences between patients with and without postoperative AKI (Table 1). Compared to patients without AKI, there was a higher prevalence of cardiovascular disease and chronic obstructive pulmonary disease (COPD) in patients who experienced AKI. This corresponded with a greater proportion of patients with an ASA class >2 in those with AKI compared to those without, indicating a higher anesthetic risk. Conversely, AKI and non-AKI patients were well-matched on age, sex, diabetes, and body mass index.

### Perioperative factors

There was no significant difference between patients with and without AKI in the use of renin-angiotensin system inhibitors, or exposure to intravenous contrast and nephrotoxic medications (Table 2). Common nephrotoxins included nonsteroidal anti-inflammatory drugs (15.4%), gentamicin or vancomycin (6.3%), and various combinations (1.7%). Preoperative hemoglobin was also not significantly different between the two groups. However, there was a greater prevalent use of loop diuretics and β-blockers, and a higher baseline urea and creatinine in patients who developed postoperative AKI (Table 2). Furthermore, patients who develop AKI were more likely to require postoperative intensive care unit admission for vasopressor support, and showed a greater decline in hemoglobin levels, and needed more blood transfusions (Table 2).

**Table 2.** Perioperative characteristics by acute kidney injury status.

Preoperative variables	All patients (*N* = 830)	No AKI (*n* = 715)	AKI (*n* = 115)	*p* Value
Renin-angiotensin blocker, *n* (%)	385 (46.4)	329 (46.0)	56 (48.7)	.59
β-blockers, *n* (%)	219 (26.4)	179 (25.0)	40 (34.8)	.028
Calcium-channel blockers, *n* (%)	208 (25.1	176 (24.7)	32 (27.8)	.47
Loop diuretics, *n* (%)	104 (12.5)	78 (10.9)	26 (22.6)	<.001
Iodinated contrast <72 h, *n* (%)^a^	62 (7.5)	53 (7.4)	9 (7.8)	.88
Nephrotoxic medications, *n* (%)	194 (23.3)	170 (23.7)	24 (20.7)	.49
Baseline urea, median (IQR) mmol/L	6.4 (5.3–8.3)	6.3 (5.2–8.1)	7.4 (5.7–9.7)	<.001
Baseline creatinine, median (IQR) µmol/L	78 (64–94)	76 (63–91)	89 (76–120)	<.001
Baseline hemoglobin, mean (*SD*) g/L	131.0 (16.8)	131.4 (16.8)	128.6 (16.8)	.10
Postoperative variables				
Intensive care unit admission, *n* (%)				
Ventilatory or other support	143 (17.2)	122 (17.1)	21 (18.3)	<.001
Vasopressor support	57 (6.9)	36 (5.0)	21 (18.3)	
Peak urea, median (IQR) mmol/L	6.7 (5.2–9.0)	6.4 (5.1–8.2)	10.8 (8.9–13.8)	<.001
Peak creatinine, median (IQR), µmol/L	83 (67–109)	79 (65–96)	140 (118–188)	<.001
Postoperative hemoglobin, mean (*SD*), g/L	113.5 (18.9)	114.5 (18.9)	107.7 (18.1)	<.001
Decline in hemoglobin, mean (*SD*), g/L	17.5 (15.1)	16.9 (14.5)	20.9 (17.9)	.008
Red blood cell transfusion, *n* (%)				
None	792 (95.4)	687 (96.1)	105 (91.3)	.019^b^
1–3 units	29 (3.5)	23 (3.2)	6 (5.2)	
>3 units	9 (1.1)	5 (0.7)	4 (3.5)	

^a^Includes intravenous and intra-arterial contrast.

^b^Fisher’s exact test.

### Intraoperative hypotension

**Figure 2.**
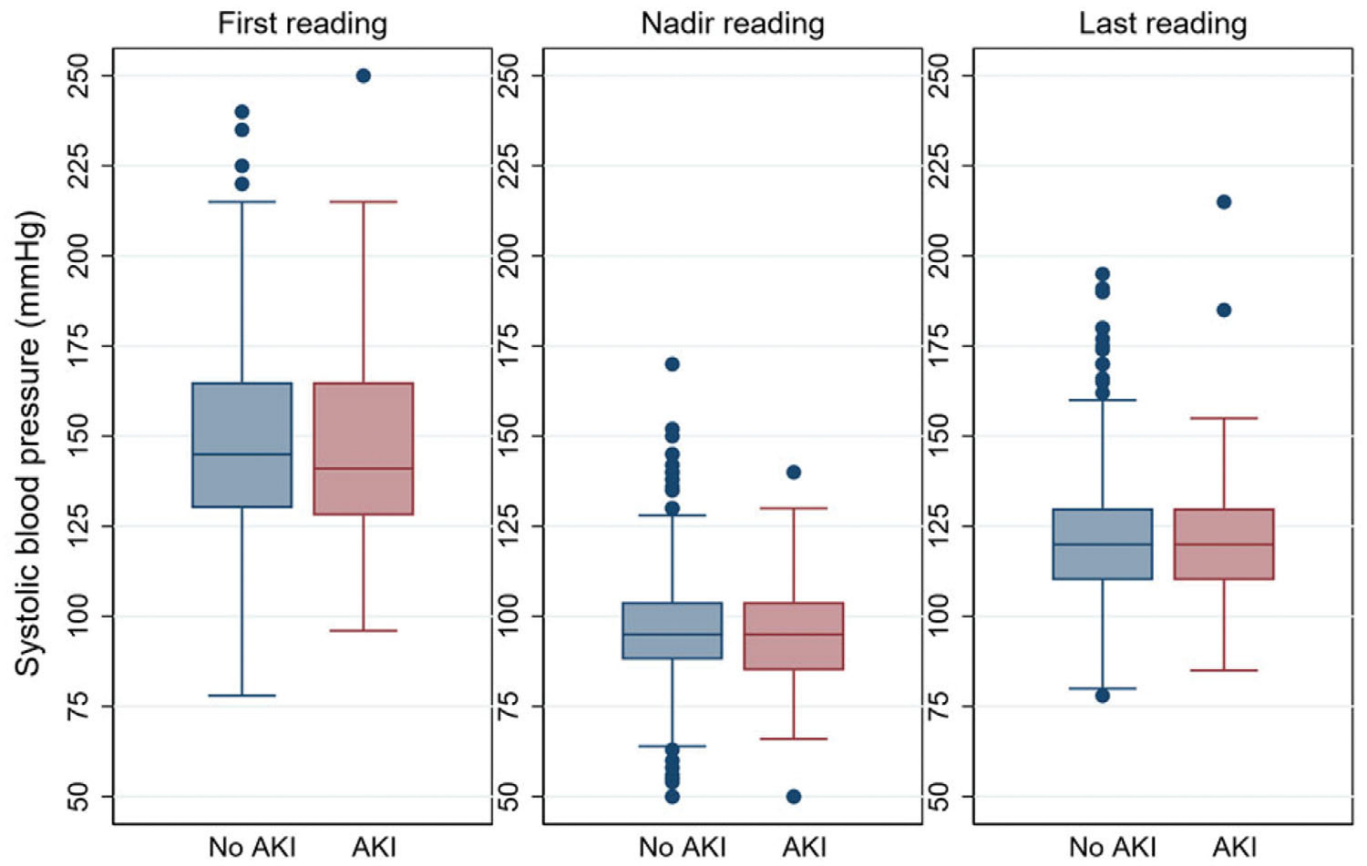
Boxplots of intraoperative systolic blood pressure readings at the beginning of surgery (first), lowest blood pressure reading ever achieved (nadir), and the final reading on the anesthetic chart (last), demonstrating that all patients drop their blood pressures intraoperatively. The nadir blood pressures offer an explanation why 85% of patients received vasopressors. Abbreviation: AKI: acute kidney injury.

Overall, the distribution of surgery duration was not significantly different between patients with AKI and patients without AKI (*p* = .65). The mean SBPs and mean MAPs were comparable at the beginning and conclusion of surgery between the two groups. The average final SBP was 25 mmHg lower, and the final MAP was 15 mmHg lower, than the initial values of these parameters (Table 3). It was clear that all patients drop their blood pressure intraoperatively, and many had nadir blood pressure levels below the hypotensive threshold (Figure 2). An episode of hypotension occurred at least once in 25.9% of patients based on SBP criteria and 11.9% based on MAP criteria. Very few patients experienced more than one episode of hypotension by either criterion. Most episodes of SBP hypotension were short, with 75% lasting <10 min (244 of 324 episodes) and only 1% of SBP hypotensive episodes lasting >20 min (3 of 324 episodes). Similarly, 67% of MAP hypotension episodes lasted <10 min (78 of 117 episodes) and 9% lasted >20 min. The only hemodynamic parameter significantly associated with postoperative AKI was the total duration of SBP hypotension, with the most obvious difference noted for those with SBP hypotension >20 min (Table 3).

**Table 3.** Intraoperative blood pressure, hypotension, and vasopressor use.

	All patients (*N* = 830)	No AKI (*n* = 715)	AKI (*n* = 115)	*p* Value
**Systolic blood pressure (SBP)**				
Initial SBP, mean (*SD*) mmHg	148 (26)	148 (26)	147 (27)	.92
Final SBP, mean (*SD*) mmHg	122 (18)	122 (18)	121 (17)	.49
Any episode of SBP < 90 mmHg, *n* (%)	215 (25.9)	184 (25.7)	31 (27.0)	.78
Duration of SBP < 90 mmHg, median (IQR) min^a^	5 (5–10)	5 (5–10)	10 (5–15)	.78
Total duration of SBP< 90 mmHg, *n* (%)				
None	615 (74.1)	531 (74.3)	84 (73.0)	.048
<10 min	116 (14.0)	102 (14.3)	14 (12.2)	
10–20 min	83 (10.0)	72 (10.1)	11 (9.6)	
>20 min	16 (1.9)	10 (1.4)	6 (5.2)	
≥2 episodes of SBP < 90 mmHg, *n* (%)	65 (7.8)	53 (7.4)	12 (10.4)	.26
Any episode of SBP < 90 mmHg ≥ 10 min, *n* (%)	60 (7.2)	49 (6.9)	11 (9.6)	.30
**Mean arterial pressure (MAP)**				
Initial MAP, mean (*SD*) mmHg	99 (17)	100 (17)	98 (16)	.27
Final MAP, mean (*SD*) mmHg	83 (12)	83 (12)	85 (11)	.29
Any episode of MAP < 60 mmHg, *n* (%)	99 (11.9)	80 (11.2)	19 (16.4)	.06
Duration of MAP < 60 mmHg, median (IQR) min^a^	5 (5–10)	5 (5–10)	5 (5–10)	.12
Total duration of MAP < 60 mmHg, *n* (%)				
None	730 (88.0)	635 (88.8)	95 (82.6)	.16^b^
<10 min	57 (6.9)	45 (6.3)	12 (10.4)	
10–20 min	32 (3.9)	25 (3.5)	7 (6.1)	
>20 min	11 (1.3)	10 (1.4)	1 (0.9)	
≥2 episodes of MAP < 60 mmHg, *n* (%)	20 (2.4)	16 (2.2)	4 (3.5)	.42
Any episode of MAP < 60 mmH*g* ≥ 10 min, *n* (%)	35 (4.2)	28 (3.9)	7 (6.0)	.28
**Vasopressors^c^**				
Duration of surgery, median (IQR) min	158 (101–241)	159 (103–240)	153 (92–248)	.65
Any vasopressor used, *n* (%)	703 (84.7)	603 (84.3)	100 (86.9)	.47
Metaraminol total dose, *n* (%)				
None	185 (22.3)	160 (22.4)	25 (21.7)	.56
0.5 to 20.0 mg	616 (74.2)	532 (74.4)	84 (73.0)	
>20.0 mg	29 (3.5)	23 (3.2)	6 (5.2)	
Ephedrine total dose*, n* (%)				
None	593 (71.5)	522 (73.0)	71 (61.7)	.011^d^
0.5 to 20.0 mg	205 (24.7)	170 (23.8)	35 (30.4)	
>20.0 mg	32 (3.9)	23 (3.2)	9 (7.8)	
Norepinephrine total dose, *n* (%)				
None	801 (96.5)	698 (97.6)	103 (89.6)	<.001^b,e^
0.5 to 20.0 mg	19 (2.3)	14 (2.0)	5 (4.4)	
>20.0 mg	10 (1.2)	3 (0.4)	7 (6.1)	
Epinephrine total dose, *n* (%)				
None	825 (99.4)	712 (99.6)	113 (98.3)	.033^b,d^
0.5 to 10.0 mg	3 (0.4)	3 (0.4)	0 (0.0)	
>10.0 mg	2 (0.2)	0 (0.0)	2 (1.7)	
Total dose of all vasopressors, *n* (%)				
None	127 (15.3)	112 (15.7)	15 (13.0)	.003^d^
0.5 to 20.0 mg	608 (73.3)	532 (74.4)	76 (66.1)	
>20.0 mg	95 (11.5)	71 (9.9)	24 (20.9)	
High dose of any vasopressor, *n* (%)^f^	68 (8.2)	47 (6.6)	21 (18.3)	<.001

^a^Average duration of hypotensive episodes, with 324 episodes of SBP <90 mmHg and 117 episodes of MAP <60 mmHg.

^b^Fisher’s exact test.

^c^Vasopressor doses reported are cumulative for the duration of surgery.

^d^Test for trend, *p* < .01.

^e^Test for trend, *p* < .001.

^f^Cumulative dose of any specific vasopressor (metaraminol, ephedrine, phenylephrine, or norepinephrine) >20 mg, or a cumulative dose of epinephrine >10 mg.

### Vasopressors

Vasopressors were commonly used intraoperatively, with 84.7% of patients (703 of 830) receiving a vasopressor at least once intraoperatively, and there was an association between vasopressor use and postoperative AKI (Table 3). The commonest vasopressor was metaraminol, followed by ephedrine, and less commonly norepinephrine. Agents rarely used included epinephrine (0.6%) and phenylephrine (0.2%). The median total dose of all vasopressors for the cohort was 5.0 mg (IQR, 1.3 to 12.8 mg), with a higher total dose used in patients who developed AKI compared to those who did not develop AKI. Except for metaraminol, there was a statistically significant association between AKI and dose of individual vasopressors as well, along with a significant test for trend (differences in AKI incidence between the two groups increase moving up categories from no vasopressors to high doses of vasopressors). Thus, compared with patients who did not receive vasopressors or received low-moderate doses, patients receiving high doses of vasopressors intraoperatively were more likely to develop AKI.

**Figure 3.**
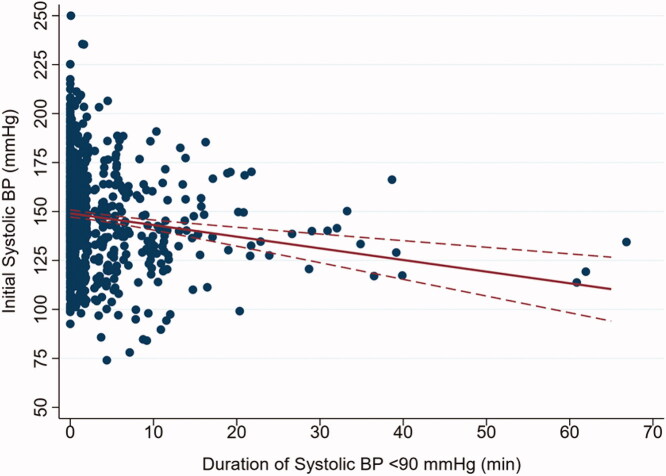
Scatterplot of initial systolic blood pressure (BP) vs. duration of systolic hypotension, with linear regression line and 95% confidence band (red lines), suggesting a weak inverse relationship between the initial blood pressure and the dose of vasopressor support used intraoperatively (*n* = 830).

Based on the scatterplot of initial SBPs versus duration of SBP hypotension (Figure 3), we further examined the hypothesis that a higher initial SBP could be associated with lower intraoperative vasopressor requirement. The results of the zero-inflated negative binomial regression of total vasopressor dose on initial SBP (per 10 mmHg) showed a statistically significant effect (*b* = −0.040, 95% CI: −0.068 to −0.011, *p* = .007). Thus, in patients who received vasopressors, a higher initial SBP was associated with lower intraoperative vasopressor requirement. We estimated that total vasopressor dose was 4% lower for every 10 mmHg increase in initial SBP.

### Logistic regression

From the initial univariable comparisons between patients with and without AKI, we examined the odds ratios from logistic regression of AKI on the relevant baseline, perioperative and intraoperative variables (Table 4). With SBP hypotension parameterized as a categorical variable, it was obvious from the odds ratios and negative test for trend (*p* = .27) that the risk of developing postoperative AKI were not significant until the cumulative duration of SBP hypotension exceeded 20 min. Thus, we used this SBP threshold to create a binary variable of SBP hypotension >20 min for testing multivariable models. There was strong evidence that the baseline serum creatinine and a significant drop in hemoglobin was associated with AKI. CKD is a well-known risk factor for AKI and is associated with increased cardiovascular disease risk. A significant hemoglobin drop is a marker severe blood loss, another known risk factor for AKI as well as hypotension. We also included the intraoperative vasopressor variables, and the postoperative vasopressor support in the 24 h postoperatively given our study includes a 48-h postoperative period. As these variables were clinically relevant, logical, and highly statistically significant, we selected them for inclusion as covariates in the initial multivariable model.

**Table 4.** Univariable logistic regression of acute kidney injury.

Variable	Odds ratio	95% CI	*p* Value
Coronary heart disease	1.95	1.26–3.00	.003
Heart failure	2.40	1.29–4.49	.006
Peripheral vascular disease	1.88	1.11–3.19	.020
Chronic hypertension	2.13	1.27–3.57	.004
Chronic obstructive pulmonary disease	1.93	1.18–3.17	.009
ASA score, per class increase^a^	1.95	1.37–2.78	<.001
Loop diuretics	2.39	1.45–3.92	.001
β-blockers	1.60	1.05–2.43	.029
Baseline creatinine, per 100 µmol/L	1.90	1.42–2.54	<.001
Chronic kidney disease	2.62	1.74–3.94	<.001
Postoperative hemoglobin, per 10 g/L	0.83	0.75–0.92	<.001
Drop in hemoglobin, per 10 g/L	1.19	1.05–1.35	.008
Red blood cell transfusion			
None	1.00	Reference	.029
1–3 units	1.71	0.69–4.29	
>3 units	5.23	1.38–19.8	
Total duration of SBP < 90 mmHg			
None	1.00	Reference	.077
<10 min	0.88	0.48–1.59	
10–20 min	0.97	0.50–1.90	
>20 min	3.79	1.35–10.7	
Total duration SBP < 90 mmHg > 20 min	3.88	1.38–10.9	.010
Any intraoperative vasopressor use	1.24	0.70–2.20	.47
High dose of any intraoperative vasopressor^b^	3.18	1.82–5.55	<.001
Total intraoperative vasopressor dose			
None	1.00	Reference	.004
0.1–20.0 mg	1.07	0.60–1.92	
>20.0 mg	2.52	1.24–5.14	
Postoperative vasopressor use	4.21	2.36–7.52	<.001
Intensive care unit admission	2.03	1.33–3.08	.001

^a^Classes 1 and 2 combined due to a low number of patients with class 1 status.

^b^Cumulative dose of any specific vasopressor (metaraminol, ephedrine, phenylephrine, or norepinephrine) >20 mg, or a cumulative dose of epinephrine >10 mg.

ASA: American Society of Anesthesiologists physical status; SBP: systolic blood pressure.

It was clear from the univariable analysis that a measure of comorbidity burden is needed in our model. In stage 1 of our multivariable analysis (Table 5), we included the modified ASA classification (classes 1 and 2 combined due to low numbers in class 1) in the multivariable model. Loop diuretics and β-blockers were dropped as they were not statistically significant after allowing for covariates and did not confound estimates. The base multivariable model (Model 1) included SBP hypotension >20 min, baseline creatinine, ASA class, hemoglobin drop, total intraoperative vasopressors, and postoperative vasopressor support. The point biserial correlation coefficient between SBP hypotension >20 min and total intraoperative vasopressor dose was 0.165, and the estimated variance inflation factors did not support collinearity. ICU admission was not included in the multivariable analysis as it was on the causal pathway to AKI, reflecting the effect of hemodynamic instability and postoperative vasopressor support on the development of AKI. After adjusting for the covariates, SBP hypotension >20 min was no longer statistically significant, with a 48% change in the *b*-coefficient. In model 2, we substituted total vasopressor dose for the high dose use of any vasopressor, with consistent negative results even though the correlation (Φ coefficient) between SBP hypotension >20 min and the high dose use of any vasopressor was 0.150, and the estimated variance inflation factor did not indicate collinearity. However, if total intraoperative vasopressors were dropped (Model 3), the estimates for SBP hypotension >20 min changed by 19% but remained not significant (*p* = .12).

**Table 5.** Multivariable logistic regression of acute kidney injury.

Model (covariates)	Odds ratio	95% CI	Δ*b*
**Stage 1**			
Total duration SBP < 90 mmHg > 20 min^a^ (univariable)	3.88	1.38–10.8	
Model 1 (SBP hypotension^a^, baseline creatinine, ASA, total intraoperative vasopressors, hemoglobin drop, postoperative vasopressors)	2.03	0.61–6.77	−48%
Model 2 (SBP hypotension^a^, baseline creatinine, ASA, high dose of any vasopressor, hemoglobin drop, postoperative vasopressors)	2.25	0.72–7.05	−42%
Model 3 (Total intraoperative vasopressors dropped from Model 1 covariates)	2.50	0.79–7.98	+19%
**Stage 2:** SBP hypotension dropped as nonsignificant	Odds ratio	95% CI	AIC/BIC
Model 4 (Total intraoperative vasopressors^a^, baseline creatinine, ASA, hemoglobin drop, postoperative vasopressors)	1.11	1.03–1.20	AIC 617.5 BIC 645.8
Model 5 (Model 4, with total intraoperative vasopressors replaced by use of high dose of any vasopressor^a^)	2.47	1.35–4.50	AIC 618.0 BIC 646.4
Model 6 (Total intraoperative vasopressors^a^, transformed creatinine, ASA, hemoglobin drop, postoperative vasopressors), dropped 1 influential case	1.11	1.03–1.19	AIC 594.1 BIC 627.1
Model 7 (Model 6, with total intraoperative vasopressors replaced by use of high dose of any vasopressor^a^)	2.47	1.34–4.58	AIC 596.2 BIC 624.5
Model 8 (Model 6, with ASA replaced by chronic obstructive pulmonary disease + chronic hypertension)	1.10	1.02–1.19	AIC 594.1 BIC 627.1
Model 9 (Model 7, with ASA replaced by chronic obstructive pulmonary disease + chronic hypertension)	2.30	1.24–4.29	AIC 594.5 BIC 627.5
Model 10 (Model 6, with transformed creatinine replaced by chronic kidney diseases status)	1.12	1.04–1.20	AIC 617.3 BIC 645.6
Model 11 (Model 7, with transformed creatinine replaced by chronic kidney disease status)	2.61	1.42–4.80	AIC 617.6 BIC 645.9

^a^Main risk factor of interest where odds ratio has been reported.

ASA: American Society of Anesthesiologists physical status classification, classes 1 and 2 were combined due to a low number of patients in class 1; Δ*b*: change in *b*-coefficient from the previous model compared; AIC: Akaike information criteria; BIC: Bayesian information criteria.

In stage 2 of the analysis (Table 5), we dropped SBP hypotension and considered total intraoperative vasopressors as the main factor of interest (Model 4). Dropping SBP hypotension from Model 1 yielded a 0.4% change in the *b*-coefficient for total intraoperative vasopressors (likelihood ratio test, *p* = .26). We also examined substituting total intraoperative vasopressor dose with the high dose use of any vasopressor (Model 5). Dropping SBP hypotension from Model 2 yielded a 5.3% change in the *b*-coefficient for high dose use of any vasopressor (likelihood ratio test, *p* = .18). Thus, we showed that SBP hypotension had no significant effect on the *b*-coefficients of these vasopressor variables and was not required in the final multivariable models.

After model diagnostics, we transformed baseline creatinine (power, −0.5) and removed one influential atypical case (extreme vasopressors, hemorrhagic shock, severe AKI) to improve model fit (Models 6 and 7). As an alternative to ASA class, the only comorbidities statistically significant in the multivariable model were COPD and hypertension (Models 8 and 9), but the model fit estimates were similar. Another option was to substitute creatinine for CKD status (Models 10 and 11), but this did not improve model fit statistics. There were no significant interactions between the covariates. Calibration plot and Hosmer–Lemeshow test showed a good fit for the data.

### Other analysis

To determine selection bias, we compared demographic and surgical data between patients who were included in the analysis and patients who were excluded. We found no significant difference in age (mean, 74.5 years vs. 75.6 years, *p* = .06) or sex (males, 53.9% vs. 51.3%, *p* = .40). However, compared to included patients, there was a smaller proportion of patients with an ASA score >2 among those excluded (67.3% vs. 56.0%, *p* = .004). Patients who were excluded also experienced a shorter duration of surgery (mean difference, 80 min; 95% CI: 63–97 min, *p* < .001). This may partly be explained by the larger proportion of excluded patients who had plastics, otolaryngology, or maxillofacial surgery (10.8% vs. 20.7%) and orthopedic surgery (11.3% vs. 23.7%), but relatively fewer general (mostly abdominal) surgery (36.9% vs. 16.5%). The differences in surgery type between included and excluded patients were statistically significant (*p* < .001).

We conducted a sensitivity analysis by excluding 26 patients who had nephrectomy surgery for urological malignancy. There was no significant change in the estimates for total intraoperative vasopressor dose (Model 6 covariates) with an OR = 1.12 (95% CI: 1.04–1.21, *p* = .005), and no significant change for high dose vasopressor use (Model 7 covariates) with an OR = 2.54 (95% CI: 1.34–4.81, *p* = .004).

## Discussion

In this study, 25.7% of elderly patients with a mean age of 75 years undergoing elective noncardiac surgery experienced at least one episode of SBP hypotension, and 7.8% experienced recurrent episodes. Most episodes of hypotension were brief, but the intraoperative use of vasopressor agents was high at 84.7%, and 11.5% received a relatively large total dose of vasopressors (>20 mg). One possible explanation for the high frequency of vasopressor and inotrope use is a preemptive approach taken by anesthetists based on declining blood pressure trends before it reached critical thresholds. We did not analyze for such trends, which may be an important consideration. A national or international study would be needed to determine if this observation was unique to our hospital network, or indeed very common practice in elective surgery. The mismatch between the frequency of documented hypotension and vasopressor use could also reflect under-documented hypotension, which is a possibility for episodes lasting <5 min which was rapidly corrected with a bolus of vasopressor agent.

The prevalence of postoperative AKI was 13.9%, of which 84.4% were categorized as KDIGO Stage 1 AKI. The only intraoperative hemodynamic parameter associated with AKI was the total duration of SBP hypotension >20 min, with an odds ratio of 3.88 (95% CI: 1.38–10.8). However, this was not independent of intraoperative vasopressor use, and was not significant after allowing for baseline kidney function, ASA class, hemoglobin drop, and postoperative vasopressor use. After adjusting for covariates in the final model, every 5 mg increase in total intraoperative vasopressor use was associated with 11% increased odds of developing AKI (95% CI: 3–19%, *p* = .006). Alternatively, intraoperative use of high dose vasopressors was associated with 2.5-fold increased odds of developing AKI (95% CI: 1.3˗fold to 4.6˗fold, *p* = .004).

Currently, the best intraoperative hemodynamic parameter and threshold to risk assess postoperative AKI is unclear. Three recent major studies provide some insight. Walsh et al. reported a study of 33,330 noncardiac surgeries, and found that even short durations of a MAP <55 mmHg was associated with postoperative AKI, and the risk incremented with greater durations of hypotension [12]. Sun et al. reported a study of 5127 noncardiac surgeries, and noted an increased risk of postoperative AKI associated with MAP <60 mmHg for >20 min, or <55 mmHg for >10 min [13]. Salmasi et al. reported a study of 57,315 surgeries, and concluded that a MAP <65 mmHg was associated with myocardial injury and AKI, and only brief exposures were required at even lower MAPs [14]. In our cohort, intraoperative MAP <60 mmHg was much less obvious, and could not be correlated to AKI risk. However, only 3.9% of our cohort had a total duration of MAP hypotension lasting ≥10 min, and only 1.3% had a total duration ≥20 min. This contrasts Sun et al., whereby 16.6% of patients recorded a MAP <60 mmHg for >10 min, and 5.3% for >20 min [13]. Walsh et al. reported 3.7% of patients recording a MAP < 55 mmHg for >20 min [12]. Salmasi et al. noted that 34.8% of patients recorded a MAP <65 mmHg for >12 min [14].

Our finding that AKI development was associated with SBP hypotension >20 min is novel. One major difference of our study compared to previous studies is the elderly population studied, with the mean age of 75 years compared with 56 to 61 years in the three large studies mentioned. Aging is associated with systolic hypertension. In elderly patients, the MAP may be a less reliable indicator for renal hypoperfusion than SBP, given the SBP hypotension was more common than MAP hypotension. The lower rates of intraoperative hypotension in our study could also be attributed to having higher proportion of patients with chronic hypertension who may have less risk of absolute hypotension. There is an argument to consider baseline blood pressure and chronic hypertension in managing intraoperative blood pressure, due to an altered autoregulation curve in chronic hypertension. In a randomized trial of elderly patients with chronic hypertension undergoing major abdominal surgery, a target intraoperative MAP of 80–95 mmHg reduced postoperative AKI at 7 days, compared to a target MAP of 65–79 mmHg or 96–110 mmHg [15]. Furthermore, some studies advocate an individualized approach by keeping intraoperative blood pressure within 10% to 20% of baseline, rather than using an absolute threshold [16].

Taking into context the studies aforementioned, our 13.9% incidence of AKI appeared high compared to the incidence of 5.6–7.4% previously reported in postoperative noncardiac surgery patients [12–14]. This was partly due to our older study population with greater cardiovascular comorbidities and baseline renal impairment. However, none of these large studies have considered vasopressor use in their analysis, which we have found to be a significant confounder, and its stronger association with AKI overshadowed that of hypotension itself. A potential reason for the greater association of AKI with vasopressor use than hypotension itself may be the use of goal-directed therapy to optimize perfusion and reduce postoperative complications, including AKI [17,18]. A previous systematic review of perioperative goal-directed therapy indicated that goal-directed fluid resuscitation *per se* did not show benefit in preventing postoperative AKI, and the greatest benefit was from inotropic support [19]. Given the high frequency of vasopressor use by our anesthetists, it was possible that we may have underestimated the frequency, severity or duration of hypotension by relying on analysis of manual recordings, which is why vasopressor use appeared to be a stronger and more reliable factor associated with the development of AKI. One previous study supported this proposition. Kheterpal et al. reported on postoperative AKI in 15,102 patients undergoing major noncardiac surgery, and determined that total vasopressor dose, vasopressor infusion, and diuretic administration, were independent risk factors for AKI [20]. However, this study excluded patients with CKD, and they used a decline in creatinine clearance from ≥80 mL/min preoperatively to <50 mL/min (up to 7 days postoperatively) as the definition of AKI.

In multivariable modeling, we selected baseline kidney function and hemoglobin drop for inclusion as they were statistically significant variables with strong clinical justification. CKD is a well-established risk factor for AKI [21]. Others have established that anemia or a decline in hemoglobin in the first 48 h were associated with AKI [22,23], and that intraoperative blood transfusions were also associated with AKI [24,25]. We confirmed similar findings in our analysis. Others have included anemia in postoperative AKI prediction tools [26]. In our study, chronic heart failure, coronary heart disease, COPD, peripheral vascular disease, hypertension, loop diuretics, and β-blockers were associated with postoperative AKI. For parsimony, we used the ASA score to represent the overall assessment of comorbidities. Others have demonstrated that ASA scores ≥4 were associated with postoperative AKI after major surgery [27]. As an alternatively to the ASA, we found that COPD and hypertension were independently associated with postoperative AKI, but the other comorbidities, loop diuretics and β-blockers were neither statistically significant nor confounders. This finding is supported by studies which demonstrated an association between AKI and chronic hypertension [28], and between AKI and COPD [20,24,29]. In terms of generalizability, our results may not be valid for patients who had undergone emergency surgery as these patients were excluded from our study. We acknowledge that practice variation exists in other countries, which may include blood pressure targets or the extent and choice of vasopressors and inotropes. These factors may influence the interpretation of our results. Whether or not specific vasopressor or inotropic agents are associated with a higher risk of AKI could be evaluated in future studies.

### Strengths and limitations

One study strength is the multisite participation within a tertiary hospital network, with broad representation of noncardiac surgery types, which could improve generalizability. We used a multivariable model to allow for confounding variables, and more importantly we accounted for vasopressor use, which most other studies did not. We incorporated the ASA classification into the modeling, which a widely used risk assessment tool for anesthetists, and almost all the information needed to determine AKI risk can be found within the anesthetic charts and documents. We also limited follow-up to 48 h postoperative, which we felt was relevant to exclude postoperative AKI due to delayed complications unrelated to intraoperative hypotension, particular sepsis complications.

In terms of limitations, there may be a selection bias created by excluding patients with missing serum creatinine. Patients undergoing elective plastics, otolaryngology, maxillofacial, and orthopedic surgery may have been underrepresented due to a higher proportion of these patients being excluded due to inadequate data to determine the primary outcome. Furthermore, these excluded patients may represent a lower risk group for AKI, given their lower ASA score and shorter duration of surgery compared to patients who were included in the analysis. In patients who did not have an admission biochemistry test, the presence of preoperative AKI could not be explicitly proven. However, patients with acute illness would normally have elective surgery postponed. Based on admission notes and anesthetist review, we assumed that all patients who proceeded to surgery were clinically well, and there was no reason to suspect AKI on admission.

Our study was also limited by its observational nature, including unclear causality inferences and unmeasured confounding. Capturing data in 5-min blocks may miss shorter episodes of hypotension and we were not able to determine equivalent fluid resuscitation in both groups. We did not include urine output data and may have underestimated the incidence of AKI. We did not estimate the effect of relative blood pressure reductions and relied on absolute thresholds. However, a previous study established that associations based on relative thresholds were not better than absolute thresholds in risk assessment [14]. There is currently no uniform definition of intraoperative hypotension, and a systematic review found that the incidence of AKI correlated with the definition of hypotension used [30]. It was unclear if our thresholds were optimal but seemed consistent with the larger studies previously conducted [16,30].

In this study of elective surgery patients, only 2% of patients received preemptive invasive (intra-arterial) blood pressure monitoring. Thus, our findings may not be generalized to services who have a much higher proportion of patients who receive invasive monitoring. We did not specifically determine if patients transitioned from noninvasive intermittent monitoring to invasive continuous monitoring during surgery but relied on the standard anesthetic chart for blood pressure recordings.

## Conclusions

High doses of intraoperative vasopressor use were independently associated with postoperative AKI in elderly patients undergoing noncardiac surgery and showed a stronger association with AKI than intraoperative hypotension identified from standard anesthetic charts recording blood pressure at 5 min intervals, at a threshold of MAP <60 mmHg and SBP <90 mmHg. The use of vasopressor variables to predict postoperative AKI needs to be validated but could improve our ability to identify high risk individuals in the perioperative period. For clinical practice, we suggest examining the anesthetic charts for vasopressor use when considering the differential diagnosis of postoperative AKI, even if the documented duration of hypotension appeared brief in the charts. For future research, we recommend including vasopressor variables in risk modeling for AKI to see if they improve prediction.

## Data Availability

Deidentified data pertaining to specific analysis may be available upon reasonable request from the corresponding author, pending approval from Monash Health research directorate.

## References

[1] Gu WJ, Hou BL, Kwong JSW, et al. Association between intraoperative hypotension and 30-day mortality, major adverse cardiac events, and acute kidney injury after non-cardiac surgery: a meta-analysis of cohort studies. Int J Cardiol. 2018;258:68–73. 29429638 10.1016/j.ijcard.2018.01.137

[2] Wijnberge M, Schenk J, Bulle E, et al. Association of intraoperative hypotension with postoperative morbidity and mortality: systematic review and Meta-analysis. BJS Open. 2021;5(1):zraa018. 10.1093/bjsopen/zraa018 PMC7893468 33609377

[3] Hobson C, Ozrazgat-Baslanti T, Kuxhausen A, et al. Cost and mortality associated with postoperative acute kidney injury. Ann Surg. 2015;261(6):1207–1214. 24887982 10.1097/SLA.0000000000000732 PMC4247993

[4] Grams ME, Sang Y, Coresh J, et al. Acute kidney injury after major surgery: a retrospective analysis of veterans health administration data. Am J Kidney Dis. 2016;67(6):872–880. 26337133 10.1053/j.ajkd.2015.07.022 PMC4775458

[5] Bihorac A, Yavas S, Subbiah S, et al. Long-term risk of mortality and acute kidney injury during hospitalization after major surgery. Ann Surg. 2009;249(5):851–858. 19387314 10.1097/SLA.0b013e3181a40a0b

[6] Turan A, Cohen B, Adegboye J, et al. Mild acute kidney injury after noncardiac surgery is associated with long-term renal dysfunction: a retrospective cohort study. Anesthesiology. 2020;132(5):1053–1061. 31929326 10.1097/ALN.0000000000003109

[7] Dai X, Hummel SL, Salazar JB, et al. Cardiovascular physiology in the older adults. J Geriatr Cardiol. 2015;12(3):196–201. 26089840 10.11909/j.issn.1671-5411.2015.03.015 PMC4460159

[8] Kellum JA, Lameire N, Aspelin P, et al. Kidney disease: improving global outcomes (KDIGO) acute kidney injury work group. KDIGO clinical practice guideline for acute kidney injury. Kidney Int Suppl. 2012;2(1):1–138.

[9] American Society of Anesthesiologists. ASA Physical Status Classification System [updated 2020 Dec 13; cited 2021 Nov 22; Published 2014]. Available from: https://www.asahq.org/standards-and-guidelines/asa-physical-status-classification-system

[10] Levey AS, Stevens LA, Schmid CH, et al. A new equation to estimate glomerular filtration rate. Ann Intern Med. 2009;150(9):604–612. 19414839 10.7326/0003-4819-150-9-200905050-00006 PMC2763564

[11] Abhayaratna WP, Smith WT, Becker NG, et al. Prevalence of heart failure and systolic ventricular dysfunction in older Australians: the Canberra heart study. Med J Aust. 2006;184(4):151–154. 16489896 10.5694/j.1326-5377.2006.tb00173.x

[12] Walsh M, Devereaux PJ, Garg AX, et al. Relationship between intraoperative mean arterial pressure and clinical outcomes after noncardiac surgery: toward an empirical definition of hypotension. Anesthesiology. 2013;119(3):507–515. 23835589 10.1097/ALN.0b013e3182a10e26

[13] Sun LY, Wijeysundera DN, Tait GA, et al. Association of intraoperative hypotension with acute kidney injury after elective noncardiac surgery. Anesthesiology. 2015;123(3):515–523. 26181335 10.1097/ALN.0000000000000765

[14] Salmasi V, Maheshwari K, Yang D, et al. Relationship between intraoperative hypotension, defined by either reduction from baseline or absolute thresholds, and acute kidney and myocardial injury after noncardiac surgery: a retrospective cohort analysis. Anesthesiology. 2017;126(1):47–65. 27792044 10.1097/ALN.0000000000001432

[15] Wu X, Jiang Z, Ying J, et al. Optimal blood pressure decreases acute kidney injury after gastrointestinal surgery in elderly hypertensive patients: a randomized study: optimal blood pressure reduces acute kidney injury. J Clin Anesth. 2017;43:77–83. 29055803 10.1016/j.jclinane.2017.09.004

[16] Wesselink EM, Kappen TH, Torn HM, et al. Intraoperative hypotension and the risk of postoperative adverse outcomes: a systematic review. Br J Anaesth. 2018;121(4):706–721. 30236233 10.1016/j.bja.2018.04.036

[17] Giglio M, Dalfino L, Puntillo F, et al. Hemodynamic goal-directed therapy and postoperative kidney injury: an updated meta-analysis with trial sequential analysis. Crit Care. 2019;23(1):232. 31242941 10.1186/s13054-019-2516-4 PMC6593609

[18] Grocott MP, Optimisation Systematic Review Steering Group, Dushianthan A, et al. Perioperative increase in global blood flow to explicit defined goals and outcomes after surgery: a cochrane systematic review. Br J Anaesth. 2013;111(4):535–548. 23661403 10.1093/bja/aet155

[19] Prowle JR, Chua HR, Bagshaw SM, et al. Clinical review: volume of fluid resuscitation and the incidence of acute kidney injury – a systematic review. Crit Care. 2012;16(4):230. 22866958 10.1186/cc11345 PMC3580679

[20] Kheterpal S, Tremper KK, Englesbe MJ, et al. Predictors of postoperative acute renal failure after noncardiac surgery in patients with previously normal renal function. Anesthesiology. 2007;107(6):892–902. 18043057 10.1097/01.anes.0000290588.29668.38

[21] Hsu CY, Ordonez JD, Chertow GM, et al. The risk of acute renal failure in patients with chronic kidney disease. Kidney Int. 2008;74(1):101–107. 18385668 10.1038/ki.2008.107 PMC2673528

[22] Walsh M, Garg AX, Devereaux PJ, et al. The association between perioperative hemoglobin and acute kidney injury in patients having noncardiac surgery. Anesth Analg. 2013;117(4):924–931. 24023017 10.1213/ANE.0b013e3182a1ec84

[23] Fowler AJ, Ahmad T, Phull MK, et al. Meta-analysis of the association between preoperative anaemia and mortality after surgery. Br J Surg. 2015;102(11):1314–1324. 26349842 10.1002/bjs.9861

[24] Teixeira C, Rosa R, Rodrigues N, et al. Acute kidney injury after major abdominal surgery: a retrospective cohort analysis. Crit Care Res Pract. 2014;2014:1–8. 10.1155/2014/132175 PMC3955689 24719758

[25] Iyigun M, Aykut G, Tosun M, et al. Perioperative risk factors of acute kidney injury after non-cardiac surgery: a multicenter, prospective, observational study in patients with low grade American society of anesthesiologists physical status. Am J Surg. 2019;218(3):457–461. 30739741 10.1016/j.amjsurg.2019.01.031

[26] Park S, Cho H, Park S, et al. Simple postoperative AKI risk (SPARK) classification before noncardiac surgery: a prediction index development study with external validation. J Am Soc Nephrol. 2019;30(1):170–181. 30563915 10.1681/ASN.2018070757 PMC6317608

[27] Abelha FJ, Botelho M, Fernandes V, et al. Determinants of postoperative acute kidney injury. Crit Care. 2009;13(3):R79. 19463152 10.1186/cc7894 PMC2717442

[28] Zhang Y, Jiang L, Wang B, et al. Epidemiological characteristics of and risk factors for patients with postoperative acute kidney injury: a multicenter prospective study in 30 Chinese intensive care units. Int Urol Nephrol. 2018;50(7):1319–1328. 29480442 10.1007/s11255-018-1828-7

[29] Pourafkari L, Arora P, Porhomayon J, et al. Acute kidney injury after non-cardiovascular surgery: risk factors and impact on development of chronic kidney disease and long-term mortality. Curr Med Res Opin. 2018;34(10):1829–1837. 29613817 10.1080/03007995.2018.1459527

[30] Bijker JB, van Klei WA, Kappen TH, et al. Incidence of intraoperative hypotension as a function of the chosen definition: literature definitions applied to a retrospective cohort using automated data collection. Anesthesiology. 2007;107(2):213–220. 17667564 10.1097/01.anes.0000270724.40897.8e

